# High-Flow Nasal Cannula Application After Extubation in Acute Respiratory Failure Patients

**DOI:** 10.3390/jcm14093087

**Published:** 2025-04-29

**Authors:** Wen-Chi Chao, Shen-Yung Wang, Chang-Yi Lin, Hou-Tai Chang, Wen-Lin Su, Chien-Hua Tseng, Kuang-Yao Yang, Shih-Chi Ku, Kuo-Chin Kao, Chieh-Jen Wang

**Affiliations:** 1 Department of Critical Care Medicine, MacKay Memorial Hospital, Taipei 104217, Taiwan; 2 Department of Medicine, Mackay Medical College, New Taipei City 252005, Taiwan; 3 Division of Pulmonary and Critical Care Medicine, Department of Medicine, MacKay Memorial Hospital, Taipei 104217, Taiwan; 4 Department of Critical Care Medicine, Far Eastern Memorial Hospital, New Taipei City 220216, Taiwan; 5 Division of Pulmonary and Critical Care Medicine, Department of Internal Medicine, Taipei Tzu Chi Hospital, Buddhist Tzu Chi Medical Foundation, New Taipei City 231016, Taiwan; 6 Division of Pulmonary Medicine, Department of Internal Medicine, Shuang Ho Hospital, Taipei Medical University, New Taipei City 235041, Taiwan; 7 Department of Chest Medicine, Taipei Veterans General Hospital, Taipei 112201, Taiwan; 8 Department of Internal Medicine, National Taiwan University Hospital and College of Medicine, Taipei 100, Taiwan; 9 Department of Thoracic Medicine, Chang Gung Memorial Hospital, Taoyuan 333, Taiwan

**Keywords:** high-flow nasal cannula, oxygen therapy, respiratory failure

## Abstract

**Background:** The optimal timing of high-flow nasal cannula (HFNC) application in acute respiratory failure patients remains uncertain. This study aimed to investigate the impact of HFNC on the outcomes of patients with acute respiratory failure, focusing on its use after extubation. **Methods:** This multicenter retrospective study enrolled adult acute respiratory failure patients requiring invasive mechanical ventilation during the first major outbreak of the COVID-19 pandemic in Taiwan from April to July 2021. Endpoints included prognosis after extubation as 28-day post-extubation mortality. **Results:** Among the patients, 107 received HFNC before intubation and 461 received conventional oxygen therapy (COT). Pre-intubation HFNC failure did not significantly affect hospital mortality but was associated with prolonged durations of mechanical ventilation and intensive care unit stay. Among 375 patients who underwent planned extubation, 158 received post-extubation HFNC and 217 received COT. HFNC application after extubation was associated with significantly reduced post-extubation 28-day mortality compared with COT. **Conclusions:** HFNC application after extubation is associated with reduced post-extubation 28-day mortality risks in acute respiratory failure patients who received planned extubation.

## 1. Introduction

High-flow nasal cannula (HFNC) is a non-invasive oxygen delivery system capable of reliably providing highly concentrated oxygen by delivering heated and humidified gas at a high airflow rate. HFNC therapy has shown excellent tolerability and clinical efficacy in various aspects, including alleviating dyspnea, improving patient comfort, facilitating secretion clearance, reducing the need for endotracheal intubation, and reducing mortality rates versus conventional oxygen therapy (COT) in patients with hypoxemia [1,2,3]. The use of HFNC is associated with several physiological benefits, including improved oxygenation, the generation of a low positive end-expiratory pressure (PEEP), alveolar recruitment, and reduced dead space [4]. During the past decade, HFNC has been increasingly applied in intensive care unit (ICU) settings to treat acute hypoxemic respiratory failure and as a post-extubation oxygen strategy to minimize the rate of endotracheal reintubation [3,5,6,7]. Early adoption of HFNC may reduce the work of breathing [8] and potentially reduce the risk of patient self-inflicted lung injury due to excessive inspiratory effort [6]. Recent European Respiratory Society clinical practice guidelines have recommended the preferential use of HFNC over COT for patients with acute hypoxemic respiratory failure and non-surgical patients after extubation [9]. However, the impact of HFNC on mortality remains controversial, and the specific patient population most likely to benefit from HFNC remains to be clarified.

During the early phases of the COVID-19 pandemic, a global resource shortage posed a significant challenge [10]. Consequently, non-invasive respiratory support strategies have gained widespread use among acute hypoxemic patients with COVID-19 [11,12]. Although the evidence regarding the efficacy of HFNC in COVID-19 remains limited, the Surviving Sepsis Campaign (SSC) guidelines recommended HFNC over COT and non-invasive ventilation (NIV) to reduce the reliance on invasive mechanical ventilation (IMV) [13]. Concerns about the use of HFNC in COVID-19 pneumonia include the risk of aerosol generation and the potential for delaying intubation, which could potentially result in worse outcomes [14].

In early 2020, the Taiwan Central Epidemic Command Center (CECC) was established to coordinate resources and develop policies to prevent the spread of COVID-19. Due to strict border controls and interventions, Taiwan’s healthcare system did not face a major outbreak until May 2021 [15]. Considering the higher risk of aerosol generation with NIV, HFNC was prioritized as the first-line therapy in selected hypoxemic COVID-19 patients based on the SSC recommendations. Our study hypothesized that, by reducing inspiratory effort and unloading respiratory muscles [16], HFNC may provide an advantage over COT after extubation in patients with COVID-19-related acute respiratory failure, leading to reduced short-term mortality. Given the unique context of a relatively well-prepared healthcare system without resource shortages, our study aimed to provide valuable information for optimizing respiratory support strategies in severe COVID-19 patients by analyzing a substantial multicenter cohort during Taiwan’s first major outbreak.

## 2. Materials and Methods

### 2.1. Study Design and Patient Selection

**Figure 1:**
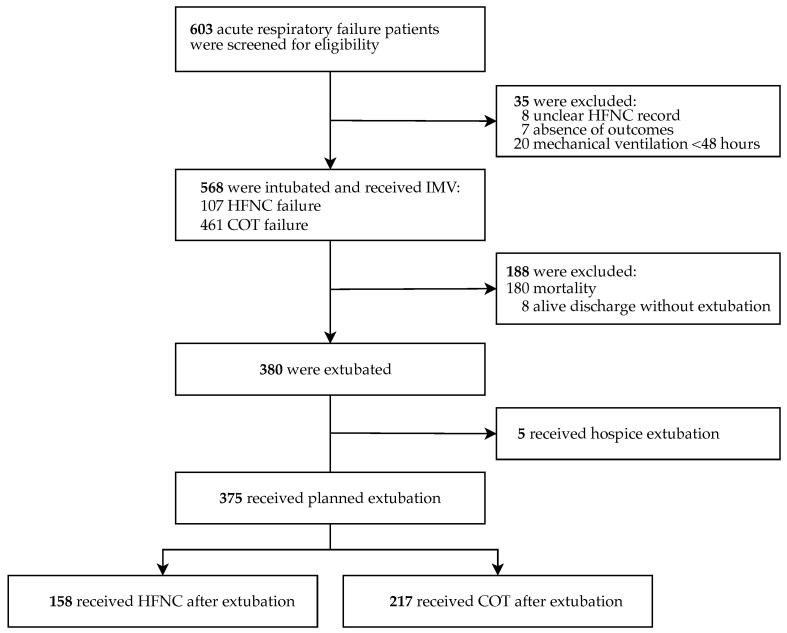
Enrollment flow chart of this retrospective study.

This study collected adult patients with COVID-19-related acute respiratory failure who required IMV during the first major outbreak in Taiwan, from April 2021 to July 2021. The retrospective cohort included patients admitted to 14 hospitals in Northern Taiwan, all confirmed by positive polymerase chain reaction testing for SARS-CoV-2 upon admission and further collected by the Taiwan Centers for Disease Control (Taiwan CDC). Medical records were reviewed by each hospital to collect clinical information, including demographic characteristics, comorbidities, laboratory results, oxygenation status, medications, clinical complications, intensive care-related parameters, and duration of hospital stay. A total of 603 patients, classified as having COVID-19-related acute respiratory failure, who had received endotracheal intubation and IMV based on the Berlin criteria [17] and the judgment of attending clinicians were screened for eligibility. The selection between HFNC and COT was at the discretion of the treating physician. A total of 35 patients were excluded: 8 due to incomplete medical records regarding HFNC use, 7 due to incomplete outcome data, and 20 who received IMV for less than 48 h. A retrospective cohort of 568 patients with documented HFNC use was included in the analysis. For the investigation of oxygenation therapies after extubation, a total of 188 patients were further excluded, including 180 mortality cases and 8 alive discharges without extubation. Among 380 patients who underwent extubation, 5 patients who had documented hospice extubation were excluded. Finally, 375 patients who had planned extubation were included in the analysis to explore the role of HFNC application after extubation in acute respiratory failure patients (Figure 1).

### 2.2. Statistical Analysis

Clinical characteristics of patients were stratified according to the use of HFNC. Continuous variables that passed the normality test were assessed using independent sample *t*-tests, while continuous variables that failed the normality test were analyzed using the independent two-group Mann–Whitney U test. Discrete variables were evaluated using the Chi-square test. Univariate Cox proportional hazards regression analysis was conducted to examine the influence of clinical demographic data and HFNC use on the prognosis of patients with acute respiratory failure. Variables with a probability value of less than 0.1 in the univariate analysis were included for further evaluation in the multivariate Cox regression model to adjust for potential confounders and identify independent prognostic predictors. The Cochran–Mantel–Haenszel log-rank test was used to compare differences in survival distributions between study groups. A two-tailed probability value of less than 0.05 was considered statistically significant. All statistical analyses described above were performed with R software version 4.4.3.

## 3. Results

A total of 568 adult COVID-19 patients with acute respiratory failure requiring IMV were included in this cohort. Table 1 shows that 107 patients (18.8%) received HFNC prior to intubation, while 461 patients (81.2%) received COT. The ages of patients who had HFNC failure before intubation were comparable to those with COT, with median ages of 69 and 68 years, respectively (interquartile range (IQR): 62–75 vs. 60–73). Both groups had a comparable male predominance (HFNC vs. COT, 62.6% vs. 65.9%), with no significant differences in body mass index (BMI) or smoking history.

Regarding comorbidities, factors such as chronic obstructive pulmonary disease (COPD), congestive heart failure (CHF), diabetes mellitus (DM), and cirrhosis did not differ significantly between the HFNC failure and COT groups. However, a significantly higher proportion of patients in the HFNC failure group had hypertension (64.5% vs. 52%, *p* = 0.027) and a higher Charlson comorbidity index (CCI) (74.8% vs. 61.6%, *p* = 0.015) versus the COT group, indicating a higher burden of comorbidity in the HFNC failure group. At the initiation of IMV, there were no statistically significant differences in lymphocyte count, lactate dehydrogenase (LDH), and ferritin levels between the two groups. However, a lower proportion of elevated procalcitonin levels was noted in patients who received HFNC before intubation (12.8%) compared with those who did not (31.4%) (*p* = 0.002), suggesting a lower proportion of severe inflammation in the HFNC failure group.

Regarding intensive care-related metrics, patients with acute physiology and chronic health evaluation (APACHE) II score ≥ 25 were less common in the HFNC failure group (11.2%) than in the COT group (23.5%, *p* = 0.008). The partial pressure of oxygen to fraction of inspired oxygen (PaO_2_/FiO_2_) ratio at IMV initiation was also lower in the HFNC failure group compared with the COT group (median: 109 vs. 139, IQR: 70–148 vs. 83–218, *p* < 0.001), suggesting potentially more primary respiratory failure in the HFNC failure group and a higher burden of organ dysfunction in the COT group. No significant differences were observed between the two groups in the respiratory rate oxygenation index (ROX) at the beginning of IMV, although there was a trend toward a lower ROX index in the HFNC failure group (5.5, *p* = 0.128), which might raise concerns about delayed intubation. The prevalence of ARDS and acute kidney injury that required renal replacement therapy (AKI-RRT) was similar between the two groups. The duration of IMV and length of ICU stay were significantly longer in the HFNC failure group. There were no significant differences in documented do-not-resuscitate (DNR) orders between the two groups. Survival analysis showed comparable in-hospital mortality between patients with failure of HFNC or COT before endotracheal intubation (log-rank test *p* = 0.400).

### HFNC Application After Extubation

The role of post-extubation HFNC was investigated in the 375 patients who underwent planned extubation. Of these, 158 patients (42.1%) received HFNC after extubation, while 217 patients (57.9%) received COT. There were no significant differences in age, gender composition, BMI, or smoking history between the two groups. Additionally, the prevalence of comorbidities such as COPD, hypertension, CHF, DM, cirrhosis, or higher comorbidity burdens (CCI ≥ 3) was similar between the two groups, as detailed in Table 2.

Patients who received HFNC after extubation had a trend towards higher proportion of tocilizumab therapy in the HFNC group (77.8%) versus the COT group (69.6%, *p* = 0.096). No significant differences were observed in the baseline intensive care profiles, including the APACHE II score ≥ 25, sedation, the use of neuromuscular blockade (NMB), ARDS, AKI-RRT, IMV duration, ICU length of stay, and the proportion of DNR orders between the two groups. Survival analysis revealed a reduced post-extubation mortality for patients who received HFNC compared with those who received COT (log-rank test *p* = 0.011).

Table 3 presents the factors associated with post-extubation mortality among the 375 COVID-19-related acute respiratory failure patients who underwent planned extubation. Aged 65 years or older, gender, a BMI over of 30 kg/m^2^, COPD, hypertension, and ARDS showed no significant association with post-extubation 28-day mortality. CHF was associated with a 6.0-fold increase in the mortality risk (95% CI, 2.081–17.243; *p* < 0.001). A CCI ≥ 3 showed a 5.1-fold increase, cirrhosis a 4.4-fold increase, and AKI-RRT a 11.5-fold increase in hazards (95% CI, 4.082–32.296; *p* < 0.001). The severity of the disease, indicated by an APACHE II score of 25 or higher, was associated with a 3.6-fold increase in risk (95% CI, 1.346–9.739; *p* = 0.011). HFNC failure before intubation did not show a significant association with post-extubation 28-day mortality (HR, 1.052; 95% CI, 0.300–3.692; *p* = 0.937). Notably, HFNC after extubation was associated with a significant reduction in post-extubation 28-day mortality (HR, 0.182; 95% CI, 0.041–0.799; *p* = 0.024).

Multivariate Cox regression analysis was performed to adjust for potential confounders. In adjusted Cox regression analyses, CHF, DM, cirrhosis, and higher CCI or APACHE II score did not show significant independent effects on post-extubation 28-day mortality (Table 4). However, AKI-RRT remained significantly associated with higher 28-day mortality risks after extubation (aHR, 11.238; 95% CI, 3.452–36.589; *p* < 0.001). Importantly, the use of HFNC after extubation continued to demonstrate a significant protective effect against mortality, even after adjusting for other factors (aHR, 0.060; 95% CI, 0.007–0.493; *p* = 0.005). These findings suggested the potential benefit of HFNC in reducing post-extubation 28-day mortality in acute respiratory failure patients with COVID-19 who received planned extubation.

## 4. Discussion

Our multicenter retrospective study revealed that failure of HFNC before intubation in critically ill COVID-19 patients requiring IMV did not affect survival outcomes compared with those without HFNC and direct intubation with mechanical ventilation. However, it was associated with prolonged duration of IMV and ICU stay. Among acute respiratory failure patients who received IMV and planned extubation, AKI-RRT was an independent factor associated with increased risks of post-extubation 28-day mortality, consistent with previous studies reporting that high hospital mortality rates ranged from 68.3% to 72.5% in hospitalized COVID-19 patients with AKI-RRT [18,19]. Notably, post-extubation HFNC was independently associated with a reduced risk of post-extubation 28-day mortality in COVID-19 patients with acute respiratory failure who received planned extubation.

The ROX index is defined as the ratio of oxygen saturation (SpO_2_)/FiO_2_ to breathing frequency and is commonly used to predict HFNC failure [20]. An initial ROX index of less than 5.26 or a persistently low ROX index during the first 12 h of HFNC was predictive of HFNC failure in critically ill COVID-19 patients, identifying those who require IMV to avoid delayed intubation [21]. In our study, the HFNC failure groups had lower initial ROX indexes compared with the COT group, suggesting potential delays in the timing of intubation in this group. Our study found that COVID-19 patients requiring invasive ventilation after HFNC failure had prolonged IMV duration and prolonged stays in ICU without a significant increase in mortality. A potential hypothesis is that, in critically ill COVID-19 patients, delayed endotracheal intubation may be associated with increased morbidity, such as prolonged ICU stay or ventilator dependence, but might not necessarily correlate with worse outcomes. A multicenter retrospective study during the early phase of the COVID-19 pandemic suggested that early intubation was associated with reduced in-hospital mortality in severe COVID-19 patients requiring intubation [22]. However, a retrospective multicenter cohort study demonstrated that initiating IMV on each day after reaching a high FiO2 threshold in COVID-19-related respiratory failure patients was associated with higher mortality versus HFNC or NIV [23]. Our result is consistent with the multicenter SOHO-COVID randomized clinical trial in France, which reported that HFNC use did not reduce 28-day mortality among patients admitted to the ICU due to COVID-19-related acute hypoxic respiratory failure [24]. Our findings indicated that patients who had HFNC failure before intubation had a lower proportion of elevated procalcitonin levels, a lower proportion of higher APACHE II scores, but a higher proportion of CCI ≥ 3, suggesting lower disease severity but a higher burden of comorbidities. Previous studies have suggested the potential of HFNC to reduce intubation rates and mortality in immunocompromised patients with respiratory failure [25,26], making HFNC a preferable option for managing immunocompromised hypoxemic COVID-19 patients. However, López-Ramírez et al. reported that HFNC exposure ≥48 h prior to IMV is associated with increased mortality [27]. Our results suggested that the failure of HFNC before intubation may not be associated with inferior survival but is associated with prolonged ICU stay and IMV duration. Due to the retrospective nature of our study, whether the use of HFNC before intubation improves or worsens patient outcomes cannot be definitively concluded, indicating further investigations.

Extubation remains a challenge among patients with acute respiratory failure [28], with approximately 10–20% of scheduled extubation leading to extubation failure, which requires reintubation in 48–72 h. This was associated with worse clinical outcomes, including prolonged use of IMV, extended ICU stay, and increased mortality [29]. Although post-extubation HFNC use may not necessarily reduce the risk of short-term mortality, it may lower the risk of endotracheal reintubation versus COT [3]. A meta-analysis conducted prior to the COVID-19 pandemic demonstrated that HFNC after planned extubation in adult ICU patients significantly reduces post-extubation respiratory failure and respiratory rates, and may increase PaO_2_ compared with COT [30]. However, data regarding HFNC as a preventive post-extubation oxygenation strategy in severe COVID-19 patients receiving IMV are limited. A small case series suggested that HFNC might have a role in weaning the patients from COVID-19-related respiratory failure [31]. Deep sedation is usually required to prevent the displacement of the endotracheal tube in critical COVID-19 patients who received IMV [32]. In addition, neuromuscular blockade agents are often used in patients with severe ARDS to prevent ventilator-induced lung injury, particularly in those undergoing prone positioning [33]. ICU-acquired weakness was reported to occur in the majority of COVID-19 patients during the awakening phase for weaning from IMV and was associated with the use of corticosteroids [34], which is a recommended treatment for patients with severe COVID-19 [35,36]. The median durations of IMV in our post-extubation study groups were both longer than 10 days (median days, HFNC 13 vs. COT 11), suggesting a higher risk of ICU-acquired weakness [37]. HFNC is increasingly recognized as an effective intervention in intensive care to prevent extubation failure and facilitate successful weaning from respiratory failure in post-extubation patients [3,38,39]. Preventive application of HFNC after extubation has been shown to reduce inspiratory effort and unload respiratory muscles, as evidenced by significantly lower esophageal pressure swings, pressure-time product, and electrical activity of the diaphragm [40]. These physiological effects have led to the hypothesis that HFNC may offer distinct advantages over COT in managing post-extubated patients. As previously mentioned, Taiwan faced its first major outbreak in May 2021, by which time landmark randomized control trials for COVID-19 management had been published [13,36,41]. Our study participants had similar disease course and received standard intensive care following international guideline recommendations. The proportions of tocilizumab and ARDS were similar in both the post-extubation HFNC group and the COT group, indicating that tocilizumab as a standard therapy for COVID-related ARDS in majority of patients did not impact post-extubation 28-day mortality. The results of our study suggested that post-extubation HFNC support might reduce extubation failure and improve short-term outcomes during the recovery phase of acute respiratory failure in COVID-19 patients. Further investigations are needed to validate the effectiveness of post-extubation HFNC in improving outcomes for severe COVID-19 patients.

Our study has several limitations primarily arising from its retrospective design, which may have affected the thoroughness of data collection. First, we lack data on patients who received HFNC and avoided IMV, which could have provided valuable insights into the effectiveness of HFNC as a NIV strategy. Second, we lack oxygenation parameters before or during HFNC use in patients who eventually required IMV; these parameters could have clarified the impact of HFNC on oxygenation and respiratory status. Third, the duration and titration of HFNC and COT before or after IMV were not available in our real-world multicenter dataset. There may be cases in the HFNC group where COT failed first, followed by the use of HFNC. Additionally, detailed information was lacking in the dataset regarding certain therapies or biomarkers associated with COVID-19-related ARDS, including vaccination, antivirals, corticosteroids, NMBs, and analgesics, which may correlate with outcomes. Our dataset also did not include information on reintubation or decisions against reintubation. Notably, among patients who were discharged alive, 12 out of 152 in the post-extubation HFNC group (7.8%) and 19 out of 201 in the post-extubation COT group (9.5%) underwent tracheostomy, potentially indicating cases of extubation failure. Finally, we were unable to determine whether the critically ill COVID-19 patients were consistently treated in the ICU or in isolation wards equipped with ICU facilities due to varying hospital policies and ICU resource allocations during the pandemic. Consequently, the recorded ICU durations may not accurately reflect the actual duration of acute critical illness, potentially affecting the interpretation of outcomes related to length of stay in these patients. Since our study specifically targets patients with acute respiratory failure caused by COVID-19, our findings may not be applicable to patients with respiratory failure due to other causes.

## 5. Conclusions

Our study suggested that patients with COVID-19-related acute respiratory failure who require ventilator support and have failed HFNC before intubation, compared with those who undergo direct intubation, do not have increased risks of in-hospital mortality. However, they experience longer stays in the ICU and more days on ventilators, which can increase the risk of complications. Therefore, in clinical practice, the risks and benefits of HFNC application prior to intubation should be carefully weighed and assessed.

In contrast, our findings suggest that the application of HFNC after planned extubation, compared with COT, may have potential benefits and reduce short-term mortality. These findings may provide valuable insights for optimizing respiratory support strategies in acute respiratory failure patients. During periods of shortages of medical resources, the use of HFNC may be prioritized for post-extubation respiratory support. Further prospective studies are warranted to explore the role and optimal timing of HFNC in the management of acute respiratory failure patients.

## Figures and Tables

**Table 1:** Patient characteristics between the failure of high-flow nasal cannula and conventional oxygen therapy before intubation.

Variable	Pre-Intubation HFNC Failure (*n* = 107)	COT (*n* = 461)	*p*-Value
**Patient profile**			
Age, years	69 (62–74.5)	68 (60–73)	0.177
Male	67 (62.6)	304 (65.9)	0.590
BMI ≥ 30	21 (20.2)	69 (15.8)	0.344
Smoking	27 (25.5)	116 (25.5)	1.000
**Comorbidities**			
COPD	8 (7.5)	24 (5.2)	0.493
Hypertension	69 (64.5)	240 (52)	0.027
CHF	10 (9.3)	43 (9.3)	1.000
DM	42 (39.3)	157 (34.1)	0.367
Cirrhosis	8 (7.5)	26 (5.6)	0.620
CCI ≥ 3	80 (74.8)	284 (61.6)	0.015
Laboratory profiles *			
Lymphocyte count, ×10^6^/L	763 (480–1091)	740 (474–1026)	0.995
Procalcitonin ≥ 0.5 ng/mL	14 (12.8)	122 (31.4)	0.002
LDH ≥ 300 U/L	73 (75.3)	310 (82.2)	0.159
Ferritin ≥ 800 ng/mL	62 (64.6)	245 (66.9)	0.754
**Intensive care-related profiles**			
APACHE II ≥ 25	12 (11.2)	116 (23.5)	0.008
PaO_2_/FiO_2_ ratio *	109.2 (70–148)	138.6 (83–218)	<0.001
ROX index *	5.5 (4.5–7.5)	6.1 (4.7–8.1)	0.128
ARDS	84 (78.5)	359 (77.9)	0.990
AKI-RRT	15 (14.0)	56 (12.3)	0.745
IMV duration, days	15 (8–27)	11 (7–21)	0.031
ICU length of stay, days	24.5 (13–34)	14 (8–27)	0.002
DNR order	38 (35.5)	161 (34.9)	0.148
Hospital mortality	41 (38.3)	164 (35.6)	0.674

Data are expressed as the median (interquartile range) or *n* (%). *: baseline at initiation of invasive mechanical ventilation.

**Table 2:** Associations between the use of post-extubation high-flow nasal cannula and patient characteristics.

Variable	Post-Extubation HFNC (*n* = 158)	COT (*n* = 217)	*p*-Value
**Patient profile**			
Age, years	66 (59–72)	65 (59–70)	0.547
Male	102 (64.6)	133 (61.3)	0.591
BMI ≥ 30	28 (18.3)	33 (15.9)	0.654
Smoking	42 (26.9)	44 (20.7)	0.200
**Comorbidities**			
COPD	5 (3.2)	10 (4.6)	0.661
Hypertension	87 (55.1)	108 (49.8)	0.367
CHF	12 (7.6)	16 (7.4)	1.000
DM	53 (33.5)	68 (31.3)	0.734
Cirrhosis	11 (7.0)	12 (5.5)	0.724
CCI ≥ 3	90 (57.0)	118 (54.4)	0.695
**Intensive care-related profiles**			
APACHE II ≥ 25	24 (15.2)	38 (17.5)	0.648
Tocilizumab	123 (77.8)	151 (69.6)	0.096
Sedation	142 (90.4)	201 (93.1)	0.470
NMB	102 (64.6)	141 (65)	1.000
ARDS	120 (75.9)	151 (69.6)	0.293
AKI-RRT	11 (7.1)	9 (4.2)	0.336
IMV duration, days	13 (8–22)	11 (7–20)	0.834
ICU length of stay, days	19 (11–32)	14 (9–27)	0.118
DNR order	23 (14.6)	35 (16.1)	0.786
**Post-extubation prognosis**			
14-day mortality	0 (0.0)	12 (5.5)	0.007
28-day mortality	2 (1.3)	14 (6.5)	0.028

Data are expressed as the median (interquartile range) or *n* (%).

**Table 3:** Univariate Cox regression analysis for post-extubation 28-day mortality.

Variable	Crude HR	95% CI	*p*-Value
Age ≥ 65	2.375	0.764–7.381	0.135
Male	0.575	0.216–1.532	0.268
BMI ≥ 30	0.383	0.050–2.927	0.355
COPD	1.610	0.213–12.191	0.645
Hypertension	1.419	0.515–3.908	0.499
CHF	5.990	2.081–17.243	<0.001
DM	2.592	0.965–6.965	0.059
Cirrhosis	4.352	1.238–15.305	0.022
CCI ≥ 3	5.051	1.145–22.274	0.032
APACHE II ≥ 25	3.620	1.346–9.739	0.011
Tocilizumab	1.109	0.358–3.440	0.857
Sedation	0.510	0.115–2.254	0.375
NMB	1.175	0.408–3.382	0.765
ARDS	5.165	0.682–39.139	0.112
AKI-RRT	11.482	4.082–32.296	<0.001
Pre-intubation HFNC failure	1.052	0.300–3.692	0.937
Post-extubation HFNC	0.182	0.041–0.799	0.024

**Table 4:** Multivariate Cox regression analysis for post-extubation 28-day mortality.

Variable	Adjusted HR	95% CI	*p*-Value
CHF	1.956	0.475–8.063	0.353
DM	1.477	0.443–4.923	0.525
Cirrhosis	2.407	0.495–11.692	0.276
CCI ≥ 3	2.559	0.498–13.144	0.260
APACHE II ≥ 25	2.251	0.719–7.044	0.163
AKI-RRT	11.238	3.452–36.589	<0.001
Post-extubation HFNC	0.060	0.007–0.493	0.009

## Data Availability

The original contributions presented in the study are included in the article. Further inquiries can be directed to the corresponding authors.
